# Breathing Out the Truth: What Fractional Exhaled Nitric Oxide Really Tells Us About Pediatric Asthma

**DOI:** 10.3390/diagnostics16111612

**Published:** 2026-05-25

**Authors:** Adriana Mihai, Ileana Katerina Ioniuc, Alina Mariela Murgu, Ancuta Lupu, Otilia Elena Frăsinariu, Elena-Lia Spoială, Eduard Vasile Rosu, Ninel Revenco, Cristina Gavrilovici

**Affiliations:** 1 Grigore T. Popa University of Medicine and Pharmacy, 700115 Iași, Romania; ileana.ioniuc@umfiasi.ro (I.K.I.); ancuta.ignat1@umfiasi.ro (A.L.); frasinariu.otilia@umfiasi.ro (O.E.F.); elena-lia.spoiala@umfiasi.ro (E.-L.S.); eduard.rosu@umfiasi.ro (E.V.R.); cri.gavrilovici@umfiasi.ro (C.G.); 2 Saint Mary’s Emergency Children’s Hospital, 700309 Iași, Romania; 3 Nicolae Testemițanu State University of Medicine and Pharmacy, MD-2004 Chișinău, Moldova; ninel.revenco@usmf.md

**Keywords:** fractional exhaled nitric oxide, FeNO, pediatric asthma, asthma diagnosis, asthma monitoring, inhaled corticosteroids

## Abstract

Asthma is the most prevalent chronic respiratory disease in childhood, and the objective assessment of airway inflammation remains a major challenge, particularly in younger children in whom conventional lung function testing is often not feasible. The aim of this narrative review is to evaluate the clinical role of fractional exhaled nitric oxide (FeNO) in pediatric asthma, focusing on its diagnostic utility, role in treatment guidance, and value in disease monitoring. A structured literature search was conducted in PubMed for studies published between January 2015 and October 2025, using predefined keywords related to FeNO, asthma, and pediatric populations. After applying the eligibility criteria, 47 studies were included in the final synthesis. Evidence from systematic reviews and clinical studies indicates that FeNO has moderate-to-good diagnostic accuracy for childhood asthma, with a pooled sensitivity of 0.79 and specificity of 0.81, and is most useful as an adjunct to clinical assessment and lung function testing. FeNO-guided therapy may reduce exacerbation rates in selected pediatric populations, although its effects on symptom control and corticosteroid use remain inconsistent. In the monitoring setting, serial FeNO measurements may provide additional information on inflammatory control, treatment adherence, and risk of future exacerbations. However, interpretation is influenced by multiple confounding factors, including atopy, allergic rhinitis, corticosteroid therapy, and asthma phenotype. In conclusion, FeNO is a valuable complementary biomarker in pediatric asthma, with particular utility in improving diagnostic and therapeutic precision. Its optimal use requires careful integration within a multimodal clinical framework rather than reliance as a standalone tool.

## 1. Introduction

Asthma is the most prevalent chronic respiratory disease in childhood and represents a significant global public health concern. Current estimates indicate that approximately 357 million individuals worldwide live with asthma, with children representing a substantial proportion of this burden [1,2]. An estimated 22 million new pediatric asthma cases are diagnosed annually, with incidence peaking in children aged 1–4 years at approximately 1885 cases per 100,000 and declining to roughly one-fifth of that rate in older adolescents aged 15–19 years [3]. Although childhood asthma mortality has declined markedly over recent decades, largely due to improved access to inhaled corticosteroids, preventable deaths continue to occur, particularly in low-resource settings [4,5].

These epidemiological patterns underscore the importance of early-life mechanisms in asthma development and highlight the clinical relevance of improving diagnostic accuracy in young children. Recurrent wheezing is common during early childhood and may reflect a heterogeneous spectrum of conditions, ranging from transient viral-induced wheeze to early manifestations of persistent asthma.

Diagnosing asthma in preschool-aged children, therefore, remains particularly challenging. Spirometry, the cornerstone of asthma diagnosis in older children and adults, requires a level of cooperation that many children under five years of age cannot reliably achieve [6]. Even when spirometry can be performed in older children, airflow obstruction may be intermittent or absent between exacerbations, potentially masking ongoing airway pathology [7].

This diagnostic uncertainty has important clinical implications. Delayed or imprecise diagnosis may result in under-treatment of children with persistent airway inflammation or, conversely, unnecessary exposure to inhaled corticosteroids in those with transient symptoms. These limitations have driven growing interest in complementary tools capable of providing objective insight into underlying airway inflammation, rather than airflow limitation alone.

Fractional exhaled nitric oxide (FeNO) has emerged as a non-invasive biomarker that reflects eosinophilic airway inflammation associated with type 2 (T2) immune pathways, commonly implicated in allergic asthma [8]. Its measurement requires only a controlled exhalation maneuver and minimal patient cooperation, making it particularly attractive for use in pediatric populations, including preschool-aged children. Reported feasibility in this age group is generally good, with success rates of approximately 80–96% from four years of age upward when age-adapted exhalation protocols are used [9,10,11], although measurement remains technically challenging below four years of age [12].

However, the interpretation and clinical utility of FeNO remain complex. Elevated FeNO values may support a diagnosis of eosinophilic asthma and predict responsiveness to inhaled corticosteroids (ICS), yet normal values do not exclude asthma, particularly in children with non-eosinophilic phenotypes or those receiving ongoing anti-inflammatory therapy [13]. In addition, FeNO levels may be influenced by atopic sensitization, allergic rhinitis, recent respiratory infections, and treatment adherence [14].

The aim of this narrative review is to critically examine the role of FeNO in pediatric asthma and to define its appropriate place within a multimodal, age-adapted approach to asthma management.

## 2. Materials and Methods

A literature search was conducted to identify studies evaluating the role of FeNO in the diagnosis and management of pediatric asthma, with particular emphasis on preschool-aged children. PubMed was searched using the following terms: (“fractional exhaled nitric oxide” OR “FeNO” OR “exhaled nitric oxide”) AND (“asthma”) AND (“child” OR “paediatric” OR “children” OR “pediatric”). The search was restricted to articles published in English, involving human subjects, and indexed between January 2015 and October 2025, in order to capture the most recent and clinically relevant evidence. Studies were included when they addressed the diagnostic, therapy-guiding, monitoring, or feasibility aspects of FeNO in pediatric populations; those limited to adult patients, animal models, in vitro experiments, or non-asthma airway disease were excluded. Relevant articles were identified through screening of titles and abstracts, followed by full-text review of potentially eligible records.

## 3. Results

**Figure 1:**
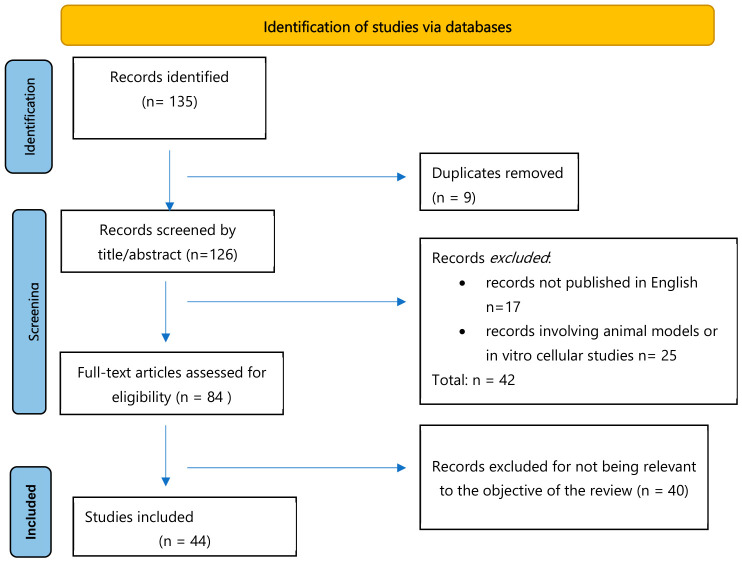
Study selection process for the identification and inclusion of studies evaluating FeNO in pediatric asthma.

The literature search identified a total of 138 records in PubMed. After removal of duplicate records (*n* = 9), the remaining 129 studies were screened based on title and abstract. During the initial screening, 42 records were excluded because they were not published in English or involved animal models or in vitro studies. Following this step, 87 articles underwent full-text evaluation. An additional 40 records were excluded due to a lack of relevance to the objectives of the review. The study selection process is illustrated in Figure 1. A total of 47 studies were ultimately included in the final synthesis. The characteristics of the included studies are summarised in Table 1.

## 4. Discussion

### 4.1. Biological Rationale of FeNO

Nitric oxide (NO) is produced within the bronchial epithelium as part of the inflammatory response, primarily through the activity of inducible nitric oxide synthase (iNOS), whose expression is upregulated by type 2 (T2) cytokines such as interleukin-4 (IL-4) and interleukin-13 (IL-13). Consequently, elevated FeNO levels reflect activation of the T2 inflammatory cascade and are closely associated with eosinophilic airway inflammation [15].

The molecular basis of this pathway has been extensively characterised. Escamilla-Gil et al. [16] mapped the complete signalling cascade, demonstrating that IL-4 and IL-13 activate the JAK/STAT-6 pathway (Janus kinase/signal transducer and activator of transcription 6), which in turn upregulates NOS2 (iNOS) [nitric oxide synthase 2 / inducible nitric oxide synthase] transcription specifically in bronchial epithelial cells. Papadopoulos et al. [17] provided further in vivo evidence by reporting that dupilumab—an anti–IL-4 receptor α monoclonal antibody—significantly reduces FeNO levels in children, confirming the IL-4/IL-13 → iNOS → FeNO axis in a clinical setting. An additional biochemical dimension involves the L-arginine → NOS → NO biosynthetic pathway, in which T2 cytokines also upregulate arginase, competing with iNOS for the L-arginine substrate.

The relationship between FeNO and asthma was first clearly established by Dupont et al. [18], who demonstrated significantly higher FeNO levels in steroid-naïve patients with mild asthma compared with healthy controls, inversely correlated with airway hyperresponsiveness. ICS treatment reduced FeNO levels to values comparable with those in healthy individuals, providing early evidence of the steroid responsiveness of this biomarker.

Konradsen et al. [20], in a cohort of 96 school-aged Swedish children with persistent asthma, demonstrated that those with both elevated FeNO and high blood eosinophils had significantly worse asthma control (*p* = 0.035), more frequent exacerbations (*p* = 0.004), and reduced FEV_1_/FVC ratios (forced expiratory volume in 1 second / forced vital capacity) (*p* = 0.001). Di Cicco et al. [19] confirmed a moderate-to-good correlation between FeNO and eosinophil counts in blood, sputum, and bronchoalveolar lavage fluid in atopic asthmatic children.

**Figure 2:**
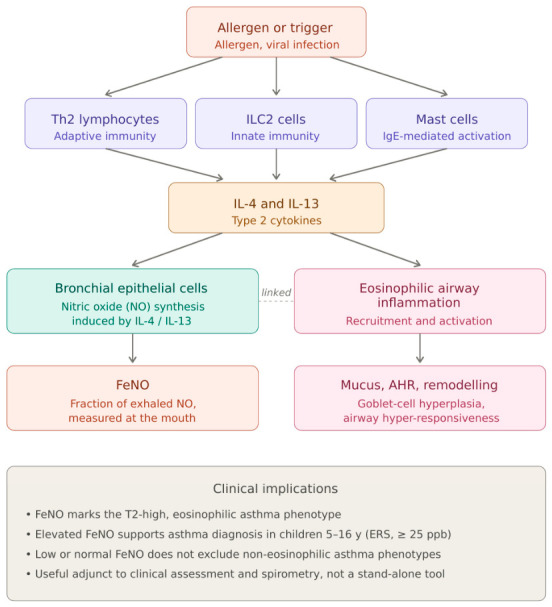
Schematic representation of the immunological pathway underlying FeNO production in type 2 airway inflammation.

It is important to recognise that FeNO reflects a specific inflammatory pathway rather than asthma as a whole. Elevated FeNO is primarily associated with T2-high, eosinophilic phenotypes and may not accurately reflect disease activity in children with non-eosinophilic or neutrophilic inflammation. The principal molecular steps of this pathway are summarised in Figure 2.

### 4.2. Use of FeNO for Asthma Diagnosis

FeNO measurement has emerged as a useful adjunct in the diagnosis of pediatric asthma, particularly in identifying eosinophilic airway inflammation. In children with asthma, FeNO levels are typically elevated compared with non-asthmatic controls, making this biomarker particularly valuable when conventional lung function tests are inconclusive or not feasible. The diagnostic evidence summarised in this section derives predominantly from cohorts of school-aged children (5–16 years), in whom validated threshold values and guideline-based recommendations—including the European Respiratory Society recommendation of ≥25 ppb—have been established. The corresponding evidence in preschool children (younger than five years) is more limited and is discussed separately in Section 4.5 (see also Table 2). Tang et al. [21] included in a systematic review eight pediatric cohorts (*n* = 2933), demonstrating a pooled sensitivity of 0.79 (95% CI 0.64–0.89) [CI = confidence interval] and specificity of 0.81 (95% CI 0.66–0.90), with an AUC of 0.87 [AUC = area under the receiver operating characteristic curve]. Wang et al. [22] reported consistent findings in a larger meta-analysis (43 studies; *n* = 13,747), noting that FeNO below 20 ppb yielded a sensitivity of 0.78 and specificity of 0.79 in the pediatric subgroup, whereas thresholds of 20–29 ppb improved specificity to 0.89. The European Respiratory Society clinical practice guideline by Gaillard et al. [23] formally recommended FeNO ≥ 25 ppb as supportive of an asthma diagnosis in children aged 5–16 years, with specificity reaching 0.99–1.00 at thresholds of 35–50 ppb.

Values above approximately 20 ppb in symptomatic children are generally considered suggestive of eosinophilic asthma [14], while values below 10–15 ppb in untreated children make eosinophilic asthma less likely [6].

Zhou et al. [24] reported that a FeNO cut-off of 25 ppb distinguished cough-variant asthma with an AUC of 0.93, sensitivity of 84.0%, and specificity of 97.1% in 115 children with chronic cough. Barāński et al. [25], in a population-based study of 449 children aged 6–10 years, found sensitivity of only 32% at 20 ppb despite a specificity of 82%, highlighting limited value as a standalone screening tool in unselected populations.

Compared with other diagnostic tools, FeNO provides complementary rather than replacement information. Spirometry remains the gold standard for demonstrating variable airflow obstruction, but is often unreliable in younger children [7]. Sputum eosinophil analysis is invasive and impractical in routine care [26]. FeNO offers a practical, non-invasive measure of airway inflammation that enhances diagnostic confidence when interpreted alongside clinical history and lung function testing. The principal threshold values reported in the pediatric literature, together with their clinical context, diagnostic or prognostic performance, and main confounders, are summarised in Table 2.

### 4.3. FeNO-Guided Therapy

The trial evidence base for FeNO-guided therapy has been generated almost exclusively in school-aged children and adolescents; consequently, FeNO-guided therapeutic algorithms cannot currently be extrapolated to children younger than five years. The use of FeNO to guide asthma therapy, particularly ICS dosing, has been investigated in several randomised controlled trials (RCTs). The rationale is that FeNO reflects underlying eosinophilic inflammation and may help tailor anti-inflammatory treatment more precisely than symptom-based strategies alone. The Cochrane systematic review by Petsky et al. [13], which pooled nine RCTs involving over 1300 pediatric patients, found that FeNO-guided management resulted in fewer children experiencing exacerbations (OR approximately 0.58) and reduced need for oral corticosteroid courses (OR approximately 0.63). These findings were corroborated by the meta-analysis of Wang et al. [29], which pooled 23 RCTs and reported a relative risk of 0.73 (95% CI 0.63–0.84) for exacerbations in FeNO-guided groups.

In contrast, the large multicentre RAACENO trial by Turner et al. [27], which randomised 509 children aged 6–15 years across 52 UK centres, found no significant benefit of adding FeNO to symptom-guided management (exacerbations: 48.2% versus 51.4%; adjusted OR 0.88 (OR = odds ratio); 95% CI 0.61–1.27; *p* = 0.49). The three-arm BATMAN trial by Voorend-van Bergen et al. [28] similarly showed no improvement in symptom-free days compared with standard care in 280 Dutch children aged 4–18 years.

When FeNO cut-offs were adjusted for atopic status, as in the dual-centre RCT by Petsky et al. [30], a substantially larger treatment effect emerged: only 22% of children in the FeNO-guided group experienced exacerbations compared with 54% in the control group (*p* = 0.021; ;number needed to treat (NNT) = 4). FeNO-guided management does not, however, consistently reduce overall ICS dose; in some studies, higher doses were required to maintain low FeNO levels [31].

These findings suggest that FeNO-guided therapy may be most useful in selected children with T2-high, eosinophilic asthma and recurrent exacerbations, whereas its value appears limited in non-eosinophilic phenotypes.

### 4.4. Use of FeNO in Asthma Monitoring

The evidence base for FeNO-based monitoring derives almost exclusively from school-aged children and adolescents, and the prognostic thresholds discussed below should not be extrapolated to preschool populations. Beyond its diagnostic role, FeNO can serve as a tool for monitoring airway inflammation and assessing treatment response. FeNO levels typically decrease with effective anti-inflammatory therapy and increase in the presence of uncontrolled inflammation or poor adherence, supporting the clinical use of serial measurements to assess inflammatory control and treatment response.

Yang et al. [32], in a prospective longitudinal study of 178 atopic asthmatic children with at least 10 serial FeNO measurements over two years, found that the highest recorded FeNO value (H-FeNO) independently predicted future loss of asthma control (adjusted OR 1.21; 95% CI 1.08–1.36), with a threshold above 47 ppb yielding a specificity of 96%. Kim et al. [33] demonstrated in 201 atopic children that combining FeNO ≥ 35 ppb with a bronchodilator response ≥ 12% provided superior prognostic accuracy compared with either measurement in isolation.

Visitsunthorn et al. [34], in a prospective cohort of 70 Thai children aged 7–20 years, identified an optimal FeNO cut-off of 31 ppb for predicting exacerbations (sensitivity 92.3%; specificity 75.4%), with no child below 20 ppb experiencing an exacerbation during 12 months of follow-up (negative predictive value 100%). Hauerslev et al. [35] demonstrated over a 5-year follow-up of 146 well-controlled Danish children that higher baseline FeNO independently predicted the need for oral corticosteroid courses (adjusted OR 1.8; 95% CI 1.0–3.2; *p* = 0.04). Cleves et al. [36] further showed that FeNO correlated with ICS adherence, supporting its use as an objective compliance marker.

However, Fielding et al. [37], in an individual patient data meta-analysis pooling 1112 children across seven RCTs, found that a 10% decline in FEV_1_ between baseline and three months predicted a 28% increased odds of exacerbation at six months, whereas a 50% rise in FeNO predicted only an 11% increase, suggesting that serial spirometric changes may carry greater prognostic weight in certain clinical contexts.

### 4.5. Limitations and Clinical Considerations

Despite its advantages, FeNO has several important limitations. Atopic conditions such as allergic rhinitis and eczema can increase FeNO independently of asthma, while ICS or systemic corticosteroids suppress FeNO levels. Transient elevations may occur during respiratory viral infections, and environmental and dietary factors contribute to additional variability in measurement.

Yoon et al. [38], in a population-based study of 933 Korean preschool children, showed that atopic allergic rhinitis alone elevated FeNO (12.43 ppb versus 8.58 ppb in non-atopic healthy controls; *p* < 0.001), potentially leading to diagnostic misclassification. Sunde et al. [39] demonstrated in birth cohorts from COPSAC2000 (*n* = 411) and COPSAC2010 (*n* = 700) that FeNO was associated with asthma only in children with concurrent aeroallergen sensitisation; in non-sensitised children, asthma was associated with lower FeNO values (aGMR 0.80 [aGMR = adjusted geometric mean ratio]; *p* = 0.05; interaction *p* < 0.0001).

Children with non-eosinophilic or obesity-related asthma may have normal FeNO levels despite clinically significant disease. Fainardi et al. [40] noted that obesity-related asthma in children is characterised by T2-low, neutrophilic or paucigranulocytic inflammation and low FeNO values, with adiposity associated with asthma only in children with low FeNO (OR 1.54–1.68).

Wang et al. [41] demonstrated in the MAAS birth cohort that height was the strongest independent predictor of FeNO and developed height-based percentile charts, showing that values above the 90th percentile in symptomatic children yielded a specificity of 96% and a positive predictive value of 97% for asthma. Clinical guidelines recommend FeNO as an adjunct rather than a standalone tool [26,42].

The practical feasibility of FeNO measurement in preschool children is consistently good when age-adapted protocols are applied, with notable exceptions confined to specific methodological settings. Heijkensköld-Rentzhog et al. [9] reported an overall success rate of 81%, reaching 96% from age four upward. Crater et al. [10] demonstrated an 83% success rate with the 6-s exhalation mode in children aged 4–5 years using NIOX VERO. The markedly lower success rate of 45% reported by Vilmann et al. [12] reflects the inclusion of children from three years of age and the use of an online single-breath protocol without age-specific adaptation, with success approaching zero below four years of age.

Sayão et al. [11] showed that FeNO progressively increased from non-wheezers (5.4 ppb) to recurrent wheezers (11.2 ppb) in 423 Brazilian children aged 3–5 years; valid measurements were obtained in 92% (423/458) of preschoolers in that cohort.

Beyond the biological and physiological confounders discussed above, several real-world considerations influence the routine clinical use of FeNO in pediatric asthma. Device availability remains uneven across healthcare systems: portable, electrochemical analysers such as NIOX VERO and NObreath are now widely used in specialist centres, but access in primary care and in low- and middle-income settings is considerably more limited [42]. Per-test consumable cost, capital expenditure for the device, and reimbursement policies vary substantially between countries, and these factors directly affect whether FeNO can be offered routinely or only in selected cases. Measurement standardisation is a further practical issue: FeNO values are device-dependent and exhalation-flow-dependent, and reference intervals derived from one analyser cannot be transposed uncritically to another. Adherence to American Thoracic Society/European Respiratory Society technical recommendations, periodic device calibration, and operator training are essential prerequisites for clinically meaningful results, particularly in the pediatric setting, where age-adapted exhalation modes are required to ensure acceptable test performance.

Our review is broadly aligned with the 2025 update of the GINA Strategy Report [26], which positions FeNO as a supportive rather than a stand-alone diagnostic or therapy-guiding biomarker. It concurs with the GINA 2025 framework on three principal points: the role of FeNO as an adjunct to clinical assessment and lung function testing rather than a replacement; the inconsistent overall effect of FeNO-guided therapy on symptom control and corticosteroid exposure; and the multiple confounders—atopy, allergic rhinitis, corticosteroid therapy, recent respiratory infection, and obesity-related phenotype—that limit interpretation. Conversely, the literature reviewed here suggests two areas in which the framework may be further refined for the pediatric population: first, the increasingly robust evidence on FeNO feasibility from approximately four years of age, supporting an extension of formal age-stratified recommendations into the preschool range; second, the consistent observation that FeNO-guided therapy yields clinically meaningful benefit in selected subgroups, particularly children with T2-high eosinophilic inflammation, confirmed atopic sensitisation, recurrent exacerbations, or suspected poor adherence to inhaled corticosteroids, which could inform more granular subgroup-specific recommendations. Recently published evidence further reinforces these refinements, with the narrative review by Davis [46] reaching converging conclusions and the algorithmic study by Galant et al. [45] illustrating how FeNO combined with impulse oscillometry can refine identification of children at residual exacerbation risk despite apparent control under guideline-based management. The retrospective analysis by Wang et al. [47] in 203 asthmatic children corroborates the added value of combining FeNO with impulse oscillometry for monitoring asthma control.

## 5. Conclusions

Fractional exhaled nitric oxide represents a valuable adjunct in the diagnosis and management of pediatric asthma, particularly in identifying eosinophilic airway inflammation and supporting a more individualised approach to care. Its non-invasive nature, relative ease of use, and ability to reflect underlying inflammatory processes make it especially useful in pediatric populations, including preschool-aged children in whom conventional diagnostic tools are often limited.

In the diagnostic setting, FeNO provides complementary information to clinical assessment and lung function testing, helping to identify T2-high asthma phenotypes and increasing diagnostic confidence when interpreted in context. In disease monitoring, FeNO offers insight into airway inflammation control, treatment adherence, and the risk of future exacerbations, potentially allowing earlier therapeutic adjustments.

FeNO is reliably feasible in preschoolers from approximately four years of age onward, particularly when device- and age-specific exhalation modes are used, while measurement remains technically challenging below this threshold.

The evidence on FeNO-guided therapy is mixed and does not currently support its routine use for all children with asthma; benefits on symptom control and lung function have been inconsistent across studies. Clinically meaningful effects, particularly in reducing exacerbations, appear most plausible in selected subgroups—specifically children with T2-high, eosinophilic inflammation, confirmed atopic sensitisation, recurrent exacerbations, or suspected poor adherence to inhaled corticosteroids.

Importantly, FeNO is not a standalone diagnostic or management tool. Its interpretation is influenced by multiple factors, including atopy, concurrent respiratory infections, corticosteroid use, and underlying asthma phenotype. As such, FeNO should be integrated within a multimodal clinical framework, alongside symptom evaluation, lung function testing, and clinical judgment.

Overall, the role of FeNO in pediatric asthma lies in its ability to enhance precision in diagnosis and management rather than to replace existing tools. Future research should focus on refining age-specific reference values, optimising diagnostic thresholds, and exploring their integration with other biomarkers to better characterise disease phenotypes and guide personalised therapy in children with asthma.

## Figures and Tables

**Table 1:** Characteristics of the 47 studies included in the final synthesis: first author and year of publication, study design or type, population and age range, sample size, and whether the study is cited in the 2025 update of the GINA Strategy Report. ATS, American Thoracic Society; COPSAC, Copenhagen Prospective Studies on Asthma in Childhood; ERS, European Respiratory Society; GBD, Global Burden of Disease; GINA, Global Initiative for Asthma; ICS, inhaled corticosteroid; IOS, impulse oscillometry; MAAS, Manchester Asthma and Allergy Study; RCT, randomised controlled trial; WHO, World Health Organization.

Ref.	Author, Year	Study Design/Type	Population (Age Range)	Sample Size (*n*)	Cited in GINA 2025
[1]	Global Asthma Network (2022)	Epidemiological report	Children 6–7 years; adolescents 13–14 years	≈158,000	Yes
[2]	Song et al. (2022)	Systematic analysis/modelling	All ages, global	—	No
[3]	Zhao et al. (2022)	Systematic analysis (GBD 2019)	Pediatric, global	—	No
[4]	Serebrisky & Wiznia (2019)	Narrative review	Pediatric, global	—	No
[5]	WHO (2025)	Fact sheet/public-health source	All ages	—	No
[6]	Rao & Phipatanakul (2016)	Narrative review	Pediatric	—	No
[7]	Murray et al. (2017)	Population-based birth cohort (MAAS)	Symptomatic school-aged children	630	No
[8]	Silkoff et al. (2005)	Clinical study	Severe refractory asthma (mainly adult)	—	No
[13]	Petsky et al. (2016)	Cochrane systematic review (9 RCTs)	Children with asthma	>1300	Yes
[14]	Dweik et al. — ATS guideline (2011)	Clinical practice guideline	All ages	—	No
[15]	Kim et al. (2016)	Narrative review	Children with allergic airway disease	—	No
[16]	Escamilla-Gil et al. (2022)	Mechanistic/molecular review	Mixed populations	—	No
[17]	Papadopoulos et al. (2024)	Narrative review	Children with asthma	—	No
[18]	Dupont et al. (1998)	Clinical study	Adults with mild asthma	—	No
[19]	Di Cicco et al. (2021)	Narrative review	Pediatric asthma	—	No
[20]	Konradsen et al. (2015)	Prospective cohort	School-aged Swedish children	96	No
[21]	Tang et al. (2019)	Systematic review/meta-analysis	Pediatric cohorts (8 studies)	2933	No
[22]	Wang et al. (2018)	Systematic review/meta-analysis	Mixed (43 studies)	13,747	No
[23]	Gaillard et al., ERS (2021)	Clinical practice guideline	Children 5–16 years	—	No
[24]	Zhou et al. (2019)	Cross-sectional clinical study	Children with chronic cough	115	No
[25]	Barański & Zejda (2022)	Population-based study	Children 6–10 years	449	No
[26]	GINA Strategy Report (2025)	International strategy/guideline	All ages	—	Yes (source document)
[27]	Turner et al.—RAACENO (2022)	Multicentre phase 3 RCT	Children 6–15 years (52 UK centres)	509	Yes
[28]	Voorend-van Bergen et al.—BATMAN (2015)	Three-arm RCT	Children 4–18 years (Netherlands)	280	No
[29]	Wang et al. (2020)	Systematic review/meta-analysis (23 RCTs)	Children with asthma	—	No
[30]	Petsky et al. (2015)	Dual-centre RCT (atopy-adjusted)	Children with asthma	63	No
[31]	Lu et al. (2015)	Systematic review/meta-analysis	Children with asthma	1017	No
[32]	Yang et al. (2015)	Prospective longitudinal cohort	Atopic asthmatic children	178	No
[33]	Kim et al. (2017)	Prospective cohort	Atopic asthmatic children	201	No
[34]	Visitsunthorn et al. (2017)	Prospective cohort	Thai children 7–20 years	70	No
[35]	Hauerslev et al. (2022)	5-year prospective follow-up	Danish children, mild–moderate asthma	146	No
[36]	Cleves et al. (2024)	Cross-sectional clinical study	Pediatric asthma, ICS-treated	—	No
[37]	Fielding et al. (2019)	Individual-patient-data meta-analysis (7 RCTs)	Children with asthma	1112	No
[38]	Yoon et al. (2017)	Population-based cross-sectional	Korean preschool children	933	No
[39]	Sunde et al. (2023)	Two birth cohorts (COPSAC2000 + COPSAC2010)	Children with/without sensitisation	411 + 700	No
[40]	Fainardi et al. (2022)	Narrative review	Children with obesity-related asthma	—	No
[41]	Wang R et al. (2022)	Multicentre cohort (MAAS)	Children— derivation of FeNO normal range	—	No
[42]	Khatri et al. — ATS (2021)	Clinical practice guideline	Mixed (adolescents/adults emphasised)	—	No
[9]	Heijkensköld-Rentzhog et al. (2015)	Method validation/feasibility	Preschool children	—	No
[10]	Crater et al. (2017)	Method validation/feasibility	Children 4–10 years (NIOX VERO)	—	No
[12]	Vilmann et al. (2017)	Cross-sectional clinical study	Healthy and asthmatic preschoolers	—	No
[11]	Sayão et al. (2016)	Cross-sectional clinical study	Brazilian preschoolers 3–5 years	458	No
[43]	Xiao et al. (2024)	Prospective observational cohort (IOS + FeNO)	Chinese preschoolers (171 asthmatic + 30 healthy)	201	No
[44]	Karrasch et al. (2017)	Systematic review	Mixed (paediatric + adult)	—	No
[45]	Galant & Morphew (2025)	Retrospective post-hoc analysis (decision-tree algorithm, IOS + FeNO)	Children 4–18 years, moderate-to-severe asthma	—	No (published after GINA cut-off)
[46]	Davis M.D. (2025)	Narrative review	Pediatric asthma	—	No (published after GINA cut-off)
[47]	Hu et al. (2025)	Retrospective cohort (IOS + FeNO)	Chinese asthmatic children (80 controlled + 123 uncontrolled)	203	No (published after GINA cut-off)

**Table 2:** FeNO threshold values reported in pediatric asthma, with their clinical context, diagnostic or prognostic performance, and main confounders. Abbreviations: AUC, area under the receiver-operating-characteristic curve; BDR, bronchodilator response; ERS, European Respiratory Society; NPV, negative predictive value; PPV, positive predictive value; Se, sensitivity; Sp, specificity.

FeNO Threshold	Age Group/Setting	Clinical Context	Sensitivity/Specificity	Main Confounders/Limitations	Key Reference(s)
<10–15 ppb	Untreated children, all paediatric ages	Diagnosis (rule-out)	Not formally reported; low likelihood of T2-high eosinophilic asthma	Possible non-eosinophilic phenotype; ICS exposure may suppress values	[6,14]
≥20 ppb	Symptomatic children, paediatric ages	Diagnosis (supportive)	Pooled (8 pediatric cohorts, various thresholds): Se 0.79, Sp 0.81, AUC 0.87; pediatric subgroup at the 20 ppb cut-off: Se 0.78, Sp 0.79	Influenced by atopy, allergic rhinitis, recent respiratory infection	[14,21,22]
20–29 ppb	Paediatric subgroup, meta-analysis	Diagnosis (improved specificity)	Specificity rising to 0.89 in this range	Sensitivity decreases with rising threshold	[22]
≥ 25 ppb	Children 5–16 years (ERS)	Diagnosis (guideline-recommended)	Supportive of asthma diagnosis (guideline-recommended)	Validated mainly in school-aged children; preschool data limited	[23]
≥25 ppb	Children with chronic cough	Diagnosis of cough-variant asthma	AUC 0.93; Se 84.0%; Sp 97.1%	Modest sample size (*n* = 115); single-centre data	[24]
≥31 ppb	Thai children 7–20 years	Exacerbation prediction	Se 92.3%, Sp 75.4% at the ≥31 ppb cut-off; NPV 100% at the <20 ppb cut-off	Single-centre cohort; not validated in younger paediatric subgroups	[34]
≥35 ppb + BDR ≥ 12%	Atopic asthmatic children (*n* = 201)	Prediction of loss of asthma control	Superior prognostic accuracy when combined with bronchodilator response	Combined biomarker; threshold not standalone	[33]
≥35–50 ppb	Children 5–16 years	Diagnosis (high specificity)	Specificity 0.99–1.00 at upper end of range	Low sensitivity at this range; limits use as screening tool	[23]
>47 ppb	Atopic asthmatic children, serial measurements	Monitoring/exacerbation prediction	Specificity 96% for future loss of control	Highest recorded value over time; requires repeated measurements	[32]
>50/≥25/≥20 ppb (hierarchy)	Children, hierarchical framework according to ICS-treatment status	Diagnosis (supportive) + monitoring	Threshold interpretation stratified by ICS use	Not validated as standalone diagnostic; supportive role only	[26]
Height-based > 90th centile	Symptomatic children (MAAS cohort)	Diagnosis (paediatric-specific normative model)	Specificity 96%; PPV 97% for asthma diagnosis	Requires height-adjusted normative data; not yet widely adopted	[41]

## Data Availability

No new data were created or analyzed in this study. Data sharing is not applicable to this article.
